# Comparison of the performance of two real-time fluorescent quantitative PCR kits for the detection of SARS-CoV-2 nucleic acid: a study based on large real clinical samples

**DOI:** 10.1186/s12985-022-01922-y

**Published:** 2022-11-18

**Authors:** Yiting Wang, Xuewen Li, Yifei Wang, Zheyu Tu, Jiancheng Xu, Junqi Pan, Qi Zhou

**Affiliations:** 1 Department of Laboratory Medicine, First Hospital of Jilin University, 1 Xinmin Street, Changchun, 130021 China; 2 University of Melbourne, Grattan Street, Parkville, VIC 3010 Australia; 3 Department of Pediatrics, First Hospital of Jilin University, 1 Xinmin Street, Changchun, 130021 China

**Keywords:** Severe acute respiratory syndrome coronavirus 2, Coronavirus disease 2019, Sansure kit, Maccura kit, Reverse transcription-polymerase chain reaction

## Abstract

**Background:**

The global pandemic of coronavirus disease 2019 (COVID-19) has led to the development of multiple detection kits by national manufacturers for severe acute respiratory syndrome coronavirus 2 viral nucleic acid testing. The purpose of this study is to evaluate the performance of different kits (i.e., Maccura kit and Sansure kit) in real clinical work using clinical samples, which will help with the optimization of the test kits.

**Method:**

During the past three months (March–May 2022), 1399 pharyngeal swabs from suspected COVID-19 patients have been initially screened using the Maccura kit in Jilin, China, and the test results were verified using the Sansure kit. The cycle threshold (Ct) values generated by the two kits were compared at different viral load levels. Correlation and consistency of the Ct values were investigated using Spearman correlation, Deming regression, and Bland–Altman plots. The cut-off Ct values of the Maccura kit were recalculated by referencing the result of the Sansure kit as a standard. Furthermore, another 163 pharyngeal swabs from suspected COVID-19 patients were collected to verify the new cut-off values.

**Results:**

As a result of the Maccura kit testing, 1192 positive cases and 207 suspected COVID-19 cases were verified. After re-examination by the Sansure kit, 1118 positive cases were confirmed. The difference between the Ct values provided by the two kits was statistically significant, except for the *N* gene at high viral load. The Ct values obtained from the two kits presented a linear positive correlation. The Maccura kit used new cut-off Ct values of 35.00 (*ORF1ab* gene) and 35.07 (*N* gene). Based on that, the validation pass rate for the new cut-off Ct values was 91.41%.

**Conclusion:**

Since the Maccura kit is found to have false positives in actual clinical work, recalculation of the cut-off values can reduce this occurrence. In order to improve the accuracy of the testing, laboratories should use two kits for COVID-19 testing, and the adjusting and optimizing of the kits for their situation are needed.

## Introduction

Since December 2019, coronavirus disease 2019 (COVID-19) which is caused by severe acute respiratory syndrome coronavirus 2 (SARS-CoV-2) infection, has spread rapidly around the world and become a pandemic that has posed a huge threat to human health [1]. As of May 3rd, 2022, more than 511 million people worldwide have been infected with COVID-19 and more than 6.23 million have died from this disease [2]. Thus, a rapid and accurate diagnosis is highly demanded to efficiently control the spread of COVID-19. Currently, analytical methods in the laboratory for COVID-19 pathogen diagnosis include nucleic acid testing (NAT), antigen testing, and serological testing [3]. Among them, several studies have shown that antigen and serological testing have limitations such as low sensitivity and lack of commercial reagents. In contrast, NAT is the preferred method for diagnosing COVID-19 [4]. NAT approach is mainly performed by testing human nasopharyngeal and oropharyngeal swabs to observe the presence of viral nucleic acids in vivo using reverse transcription-polymerase chain reaction (RT-PCR), reverse transcription loop-mediated isothermal amplification, and genome sequencing [5]. The National Health Commission of the People’s Republic of China has updated the COVID-19 treatment protocol several times, and RT-PCR detection of viral nucleic acid has been always used as a gold-standard indicator for diagnosis and treatment monitoring [6]. The NAT method has good sensitivity and specificity, and it requires specialized personnel, analytical instruments, and kits [7].

As the number of people infected with COVID-19 is surging worldwide, manufacturers in various countries are rapidly developing and producing kits due to the huge demand for NAT [8]. Under conventional procedures, the approval of newly developed kits is subject to a series of rigorous clinical validation and evaluation. Due to the severe situation of COVID-19 caused by the rapid transmission of the virus, the Food and Drug Administration approved the kits in an emergency. However, the kits which may not have undergone sufficient performance validation and evaluation can increase risk during clinical testing [9]. Currently, the approved NAT kits are mainly targeting three conserved gene sequences in the SARS-CoV-2 viral genome, including open reading frame 1ab (*ORF1ab*), nucleocapsid protein (*N*), and envelope protein (*E*) genes [10]. Recently, studies have shown that different brands of NAT kits have varied diagnostic efficacies [11]. Thus, laboratories should pay attention to the evaluation of the kits from different vendors and select appropriate testing protocols so that NAT kits can be better utilized in clinical settings.

The “Guidelines for the implementation of total 2019-nCoV nucleic acid detection (2nd edition)” issued by the National Health Commission of the People’s Republic of China stated that: The laboratory should choose at least two kits, one as the primary screening kit for routine testing, with a minimum detection limit of less than or equal to 500 copies/mL; the other should be more sensitive than the primary screening kit, with a minimum detection limit of 100 to 300 copies/mL, for re-testing confirmation when the primary screening is positive [12]. In view of the above, the Novel Coronavirus 2019-nCoV Nucleic Acid Detection Kits (RT-PCR method) produced by Maccura Biotechnology Co., Ltd (Sichuan, China) and Sansure Biotechnology Co., Ltd (Hunan, China) were selected for this study (abbreviation: Maccura kit, Sansure kit). The Maccura kit is a triple fluorescent PCR NAT kit for *ORF1ab*, *N*, and *E* gene targets. It uses RNA pseudovirus as an exogenous internal standard, and the main components of its enzyme mixture are Taq and UDG enzymes, and it has a minimum detection limit of 450 copies/mL. The Sansure kit targets *ORF1ab* and *N* genes. Rnase P is used as the endogenous internal standard, and the main components of its enzyme mixture are RT and Taq enzymes, with a minimum detection limit of 200 copies/mL. Both kits were authorized for clinical use in China and have received emergency use authorizations issued by the Food and Drug Administration [13]. Currently, most performance comparison studies on SARS-CoV-2 NAT kits have used characteristic reference substances and lacked analysis using a large number of clinical samples [14, 15]. To this end, this study analyzed the results of both kits on the same batch of clinical samples to evaluate the performance of the different kits in actual clinical work and to optimize the kits already in use. The purpose of this study was to provide a reference for future NAT and its application in the clinical laboratory.

## Methods

### Study subjects and data collection

The SARS-CoV-2 NAT was performed in Jilin from March to May 2022, and the standardized collection of pharyngeal swab samples was conducted by health care workers locally. The sample collection process strictly followed the “Guidelines for the implementation of regional 2019-nCoV nucleic acid detection (3rd edition)” and “Working manual of novel coronavirus nucleic acid detection for medical institutions (second trial edition)” [16, 17]. Nasopharyngeal swab collection: the sampling personnel gently supported the head of the collected person with one hand, held the swab against the nostril with the other hand, entered along the bottom of the lower nasal tract and slowly penetrated backward. When the tip of the swab reached the posterior wall of the nasopharyngeal cavity, they gently rotated for a circle and slowly removed the swab. Oropharyngeal swab collection: the sampling personnel moistened the swab in sterile saline, and the collected person tilted his head slightly, opened his mouth wide, and made an “ah” sound to reveal the pharyngeal tonsils on both sides after gargling with saline. The sampling personnel stretched the swab over the root of the tongue of the person being sampled and rubbed the pharyngeal tonsils on both sides back and forth with a slight force at least 3 times, and finally wiped up and down the posterior pharyngeal wall at least 3 times to remove the swab. The collected swab heads were dipped into sampling tubes containing 2–3 ml of virus preservation solution (containing guanidine salt), the tails were discarded, and the tubes were sealed in suitably sized plastic bags after screwing on the caps. The collected specimens were left at room temperature and sent to the laboratory for immediate testing within 2–4 h. The initial screening of 1399 suspected COVID-19 patients was performed using the Maccura kit, followed by re-evaluating the test results using the Sansure kit. The positive cases were finally reported based on the data of the Sansure kit as confirmed results. With the Sansure kit, three replicate samples were used for parallel retesting and the median values of the test results were recorded in the study. Furthermore, NAT data from 163 additional patients with suspected COVID-19 for both kits were collected as a validation cohort. This study was approved by the Ethics Committee of the First Hospital of Jilin University (No. K2022028). The requirement for written informed consent was waived owing to the retrospective nature of the study.

### Laboratory testing

RT-PCR experiments were performed in a mobile microbiological testing vehicle. RNA extraction was carried out using a nucleic acid extraction (NAE) system (SSNP-9600A, Jiangsu Bioperfectus Technologies Co., Ltd) with the accompanying NAE or purification reagents (batch number 20210935). The operation procedure strictly followed the manufacturer’s protocol. The nucleic acid amplification was performed on a real-time fluorescence quantitative PCR detection system (SLAN-96P, Shanghai Hongshi Medical Technology Co., Ltd). The PCR reaction system configuration, reaction parameters, and program settings were set based on the instructions of the two kits (Maccura and Sansure), respectively. The general information of the two kits is listed in Table 1. Quality control and assurance, including three negative and a weakly positive control, were included in each run to identify the false-negative and false-positive results.

**Table 1:** General information of the two kits

Test kits	Nucleic acid volume (μL)	Internal standard	Number of cycles	Minimum detection limit (copies/mL)	Target genes	Test result positive			
Maccura kit	20	Yes	40	450	*ORF1ab* / *N* / *E*	*ORF1ab* gene Sigmoidal amplification curve and Ct ≤ 38	*N* gene Sigmoidal amplification curve and Ct ≤ 38	*E* gene Sigmoidal amplification curve and Ct ≤ 37	
						+	+	+	positive
						+	+	−	positive
						+	−	+	positive
Sansure kit	20	Yes	45	200	*ORF1ab* / *N*	*ORF1ab* gene Sigmoidal amplification curve and Ct ≤ 40	*N* gene Sigmoidal amplification curve and Ct ≤ 40		
						+	+		positive
						+	−		positive
						−	+		positive

*Ct* Cycle threshold

### Experimental design

Based on the minimum detection limit of the kit, the collected dataset was divided into COVID-19 positive and negative groups using the results of the Sansure kit as a standard. Assessment of viral load was carried out based on cycle threshold (Ct) values obtained from Sansure kit for *N* gene amplification, which is a specific target for SARS-CoV-2. The Ct values of the *N* gene were then converted into the qualitative results of viral load [18]. Furthermore, the data in the COVID-19 positive group was divided into three subgroups: high (Ct < 25), moderate (25 ≤ Ct ≤ 30), and low viral load (Ct > 30).

### Statistical analysis

For statistical analysis of the testing results, the Shapiro–Wilk test was used to test the normality of the data. When the data was non-normally distributed, the Mann–Whitney *U* test was applied to compare the differences between groups. In addition, the Spearman correlation was used to characterize the relationship between the two Ct-value groups obtained from the two kits. Furthermore, the Deming regression was introduced to analyze the linearity of the above-mentioned results. In parallel, the Bland–Altman plot was used to analyze the agreement between the two Ct-value datasets from the two testing kits. Based on calculating the bias and standard deviation (SD), a good agreement between the two methods was considered if most points fell within the 95% limits of agreement (bias ± 1.96 SD) [19]. Besides, the receiver operating characteristic (ROC) curve and area under the curve (AUC) were used to analyze the differentiation of positive or negative patients by the Maccura kit test results. *P* < 0.05 (*) was considered a statistically significant difference. GraphPad Prism 8 (GraphPad, San Diego, California, USA) and SPSS 23.0 (IBM, Armonk, NY, USA) were used for data analysis and graphical plotting.

## Results

### Difference, correlation, and consistency analysis between the Ct values obtained from the two kits

**Fig. 1:**
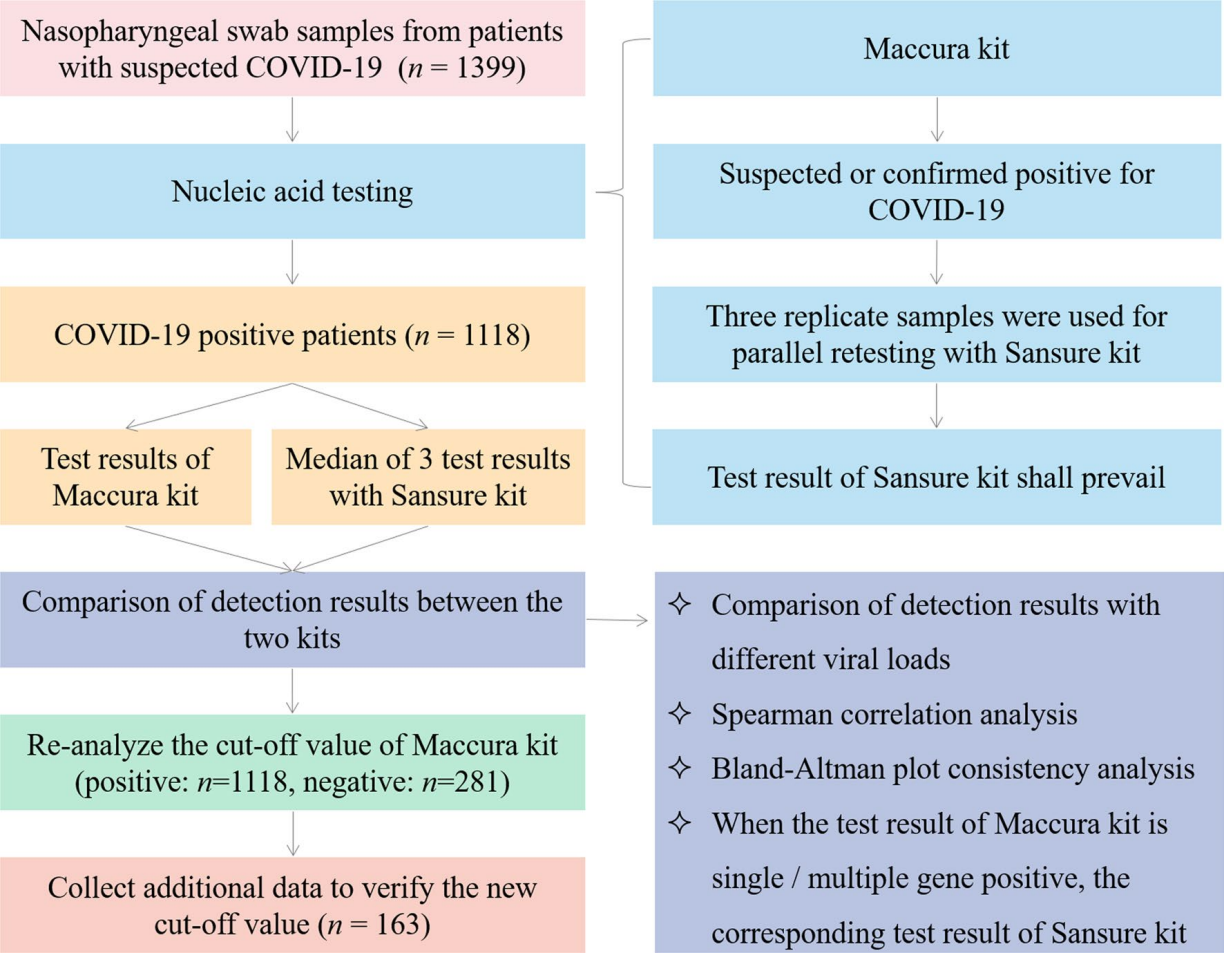
Flow chart of the study. COVID-19: Coronavirus disease 2019

**Fig. 2:**
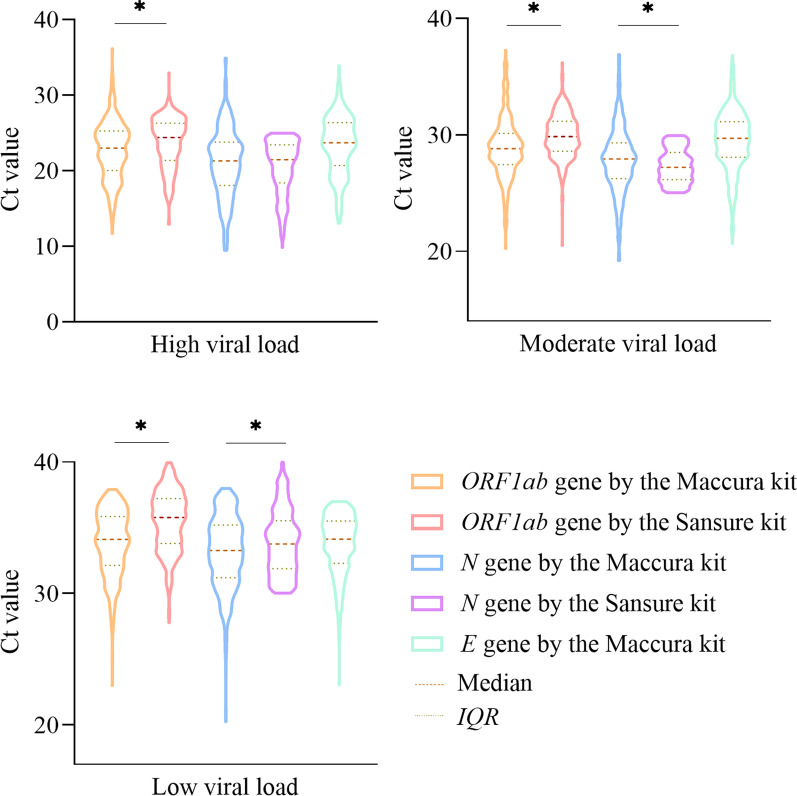
The Ct values obtained from the two kits at different viral loading levels. Ct: Cycle threshold. IQR: interquartile range. *P < 0.05

**Fig. 3:**
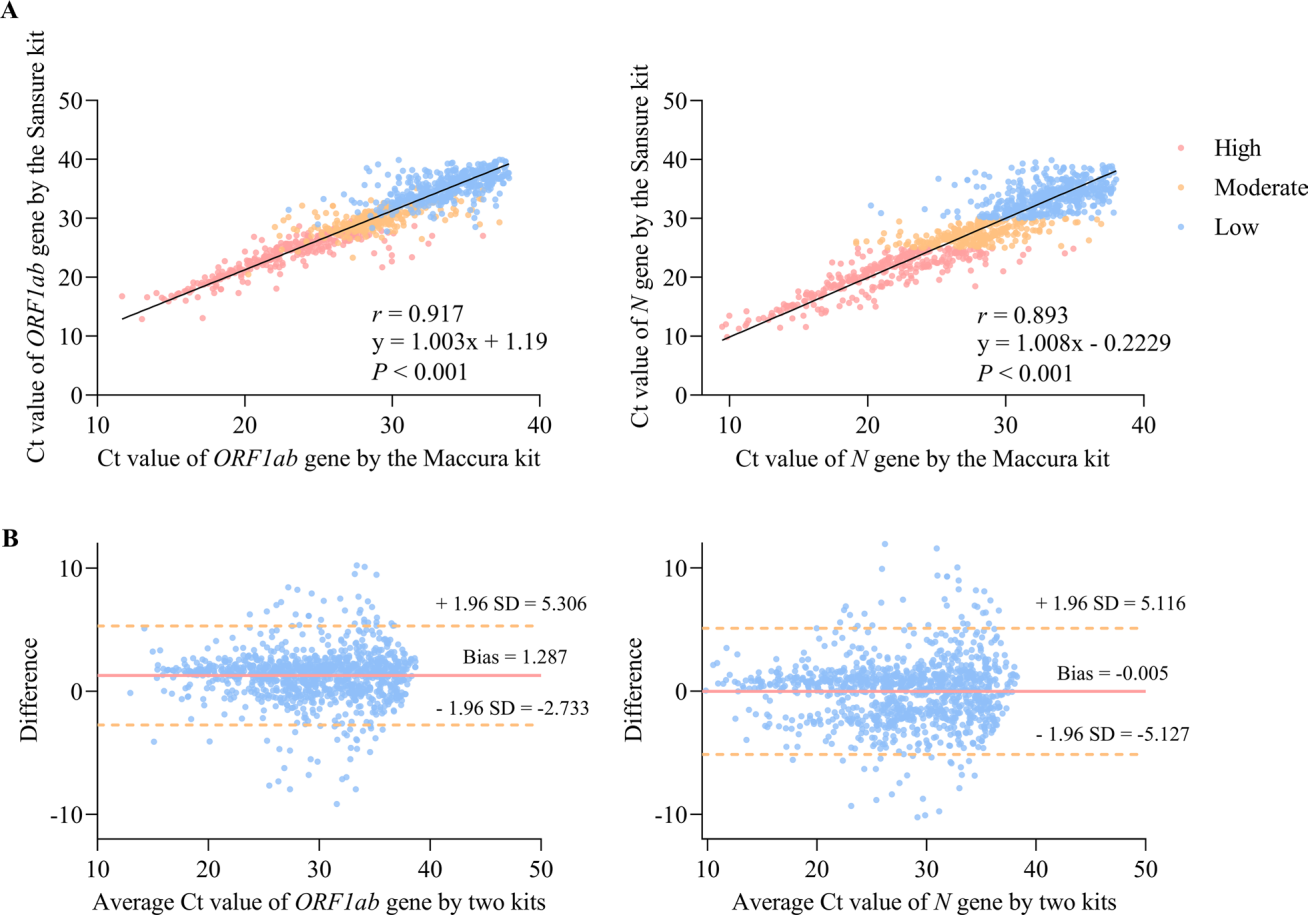
Correlation and consistency analysis between the Ct values obtained from the two kits. Ct: Cycle threshold. SD: standard deviation

The flow chart of this study is shown in Fig. 1. Among the 1399 samples tested for SARS-CoV-2, 1118 samples were positive while the other 281 were negative. The results of the Shapiro–Wilk test showed that the data were non-normally distributed with *W* values of 0.923, 0.938, 0.952, 0.967, and 0.974 (from left to right: *ORF1ab*, *N*, *E* genes of the Maccura kit, *ORF1ab*, *N* genes of the Sansure kit) (*P* < 0.05). The test results on 1399 samples from the two kits are shown in Table 2. The results of the Maccura kit assay performance were evaluated using the Sansure kit as a reference standard. As shown in Table 3, the Ct values obtained for the 1118 COVID-19 positive patients tested by the two kits were analyzed. As a result, these COVID-19 positive patients were further classified into high viral load group (*n* = 327), moderate viral load group (*n* = 291), and low viral load group (*n* = 500). The results of the Mann–Whitney *U* test showed that the Ct values of the *ORF1ab* gene amplification obtained from the Sansure kit were higher than those from the Maccura kit in the high, medium, and low viral load groups (*Z* = − 4.137, − 6.165, − 9.843, respectively). In contrast, the Ct values of the *N* gene amplification obtained from the Sansure kit were lower than those from the Maccura kit in the medium viral load group (*Z* = − 2.687), while the opposite result of *N* gene amplification was observed in the low viral load group (*Z* = − 3.169) (*P* < 0.05) (Fig. 2). The results of the Spearman correlation analysis showed that the Ct values for the amplification of *ORF1ab* and* N* genes obtained by the two kits were highly correlated (*P* < 0.001) (Fig. 3A). The Deming regression result showed that the slope of the equation for the *ORF1ab* gene with 95% confidence interval (CI) was 1.003 (0.984–1.023), and the slope of the equation for the *N* gene was 1.008 (0.984–1.031). Therefore, there was a linear relationship between the Ct values obtained from the two kits (Fig. 3A). The Bland–Altman plots showed that 6.59% (70/1062) and 5.23% (57/1090) of the Ct values of the *ORF1ab* and *N* amplification provided by the two kits fell outside the 95% limits of agreement (*ORF1ab* gene: − 2.733–5.306; *N* gene: − 5.127–5.116), with 93.41–94.77% agreement between the two kits (Fig. 3B).

**Table 2:** Results and comparison of the testing on 1399 clinical samples by the two kits

*n*		Maccura kit		All
		Positive	Negative	
Sansure kit	Positive	1072	46	1118
	Negative	120	161	281
All		1192	207	1399
Sensitivity = 1072 / (1072 + 46) × 100% = 95.89%				
Specificity = 161 / (120 + 161) × 100% = 57.30%				
Positive predictive value = 1072 / (1072 + 120) × 100% = 89.93%				
Negative predictive value = 161 / (46 + 161) × 100% = 77.78%				
Accuracy = (1072 + 161) / (1072 + 46 + 120 + 161) × 100% = 88.13%

**Table 3:** The Ct values obtained by testing 1118 COVID-19 positive patients using the two kits

Cycle threshold	Maccura kit			Sansure kit	
	*ORF1ab* gene (*n* = 1080)	*N* gene (*n* = 1090)	*E* gene (*n* = 991)	*ORF1ab* gene (*n* = 1097)	*N* gene (*n* = 1118)
Median	29.70	28.82	29.88	31.00	28.85
First Quartile	25.28	23.92	25.56	26.79	24.20
Third Quartile	33.83	32.99	33.39	35.31	33.33
Minimum	11.67	9.46	13.05	12.90	9.86
Maximum	37.93	38.00	37.00	39.95	40.00

### Re-analysis and validation of the cut-off Ct value of the Maccura kit using the Sansure kit results as a reference standard

**Fig. 4:**
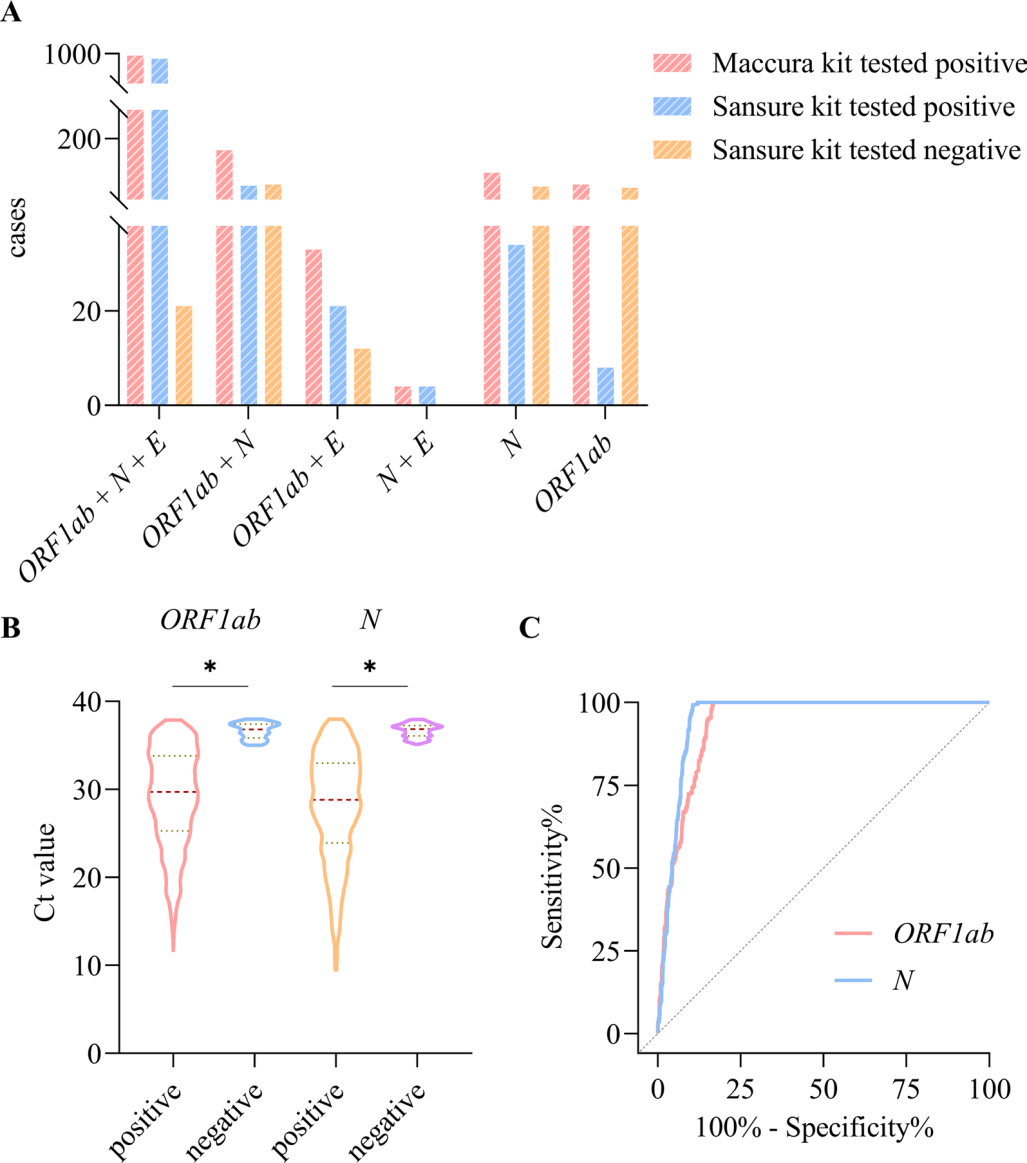
False positive and cut-off value analysis of Maccura kit. **A** The test results of the Sansure kit when 1399 patients had different test results of the Maccura kit. **B** Ct values were obtained with the Maccura kit in case of positive and negative COVID-19. **C** ROC curve. Ct: Cycle threshold. *P < 0.05

**Fig. 5:**
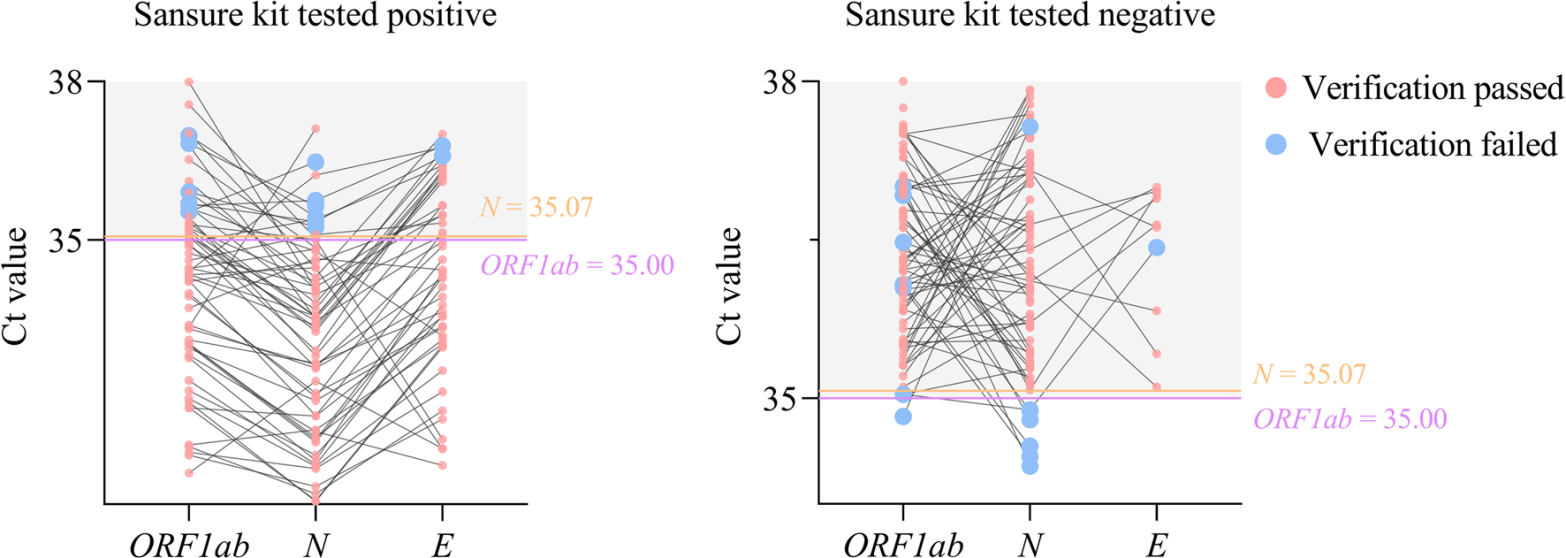
Re-evaluation of the Ct values obtained from Maccura kit using the new cut-off value. Ct: Cycle threshold

We investigated the test results of the Sansure kit, of which the results obtained from the Maccura kit were positive for multiple genes or a single gene. The testing by the Maccura kit resulted in 1192 positive cases (*ORF1ab* + *N* + *E* genes tested positive: *n* = 988, *ORF1ab* + *N* genes tested positive: *n* = 171, *ORF1ab* + *E* genes tested positive: *n* = 33). Later on, the presence of 120 false positives was found after repeating the detection with the Sansure kit. At the same time, the Maccura kit showed 207 suspected COVID-19 patients (*N* + *E* genes tested positive: *n* = 4, *ORF1ab* gene tested positive: *n* = 87, *N* gene tested positive: *n* = 116), and 46 of them were confirmed to be positive by the Sansure kit testing (Fig. 4A). As shown in Fig. 4B, the results of the Mann–Whitney *U* test showed that the Ct values of the *ORF1ab* (*Z* = − 19.67) and *N* (*Z* = − 19.86) genes amplification obtained from the Maccura kit were significantly different between the COVID-19 positive and negative groups (*P* < 0.05). The ROC curves showed that the Ct values of *ORF1ab* (AUC [95% CI] = 0.938 [0.923–0.951], Sensitivity [95%CI] = 0.831 [0.808–0.853], Specificity [95%CI] = 1 [0.982–1], *P* < 0.05) and *N* (AUC [95% CI] = 0.951 [0.938–0.962], Sensitivity [95%CI] = 0.879 [0.859–0.899], Specificity [95%CI] = 1 [0.981–1], *P* < 0.05) genes amplification obtained by the Maccura kit could clearly distinguish COVID-19 positive cases from negative ones (Fig. 4C). Thus, the new cut-off Ct values for recalculating the Maccura kit were determined (*ORF1ab* gene: Ct ≤ 35.00; *N* gene: Ct ≤ 35.07). Of the 163 cases to be validated, 62 were tested positive by the Sansure kit. The test results of the Maccura kit in 14 samples failed according to the new interpretation regulation, resulting in a validation pass rate of 91.41% (Fig. 5).

## Discussion

NAT method exhibits the advantages of promising sensitivity and specificity for viral detection. However, the accuracy of the test also depends on the sample quality, experimental factors, and kit performance [20]. In this study, we compared two commercial kits (Maccura and Sansure) for RT-PCR detection of SARS-CoV-2 nucleic acids. The Ct values obtained from the two kits were highly correlated, with a linear relationship and good agreement. Meanwhile, it was found that the difference between the two Ct-value datasets was also statistically significant, except for the Ct values of the *N* gene amplification at high viral loads.

Currently, most studies have focused on false-negative results of NAT, since it may affect the outcomes of early diagnosis and treatment [21]. Despite the low number, the false-positive results cannot be neglected [22–24]. It will mislead the self-isolation of patients, and interfere with the routine of the normal population and the treatment plan of the sick population, leading to an unnecessary burden on the health system [25]. In this study, the Sansure kit was used as a reference standard to evaluate the detecting performance of the Maccura kit. The Sansure kit was selected as the reference standard for two reasons. First, it has higher sensitivity with a minimum detection limit of 200 copies/mL, compared to the Maccura kit. Second, it has been proved as the RT-PCR kit with the best combination of sensitivity and specificity compared to the other kits [26]. In this study, the Maccura kit was found to have false-positive results with low specificity (57.3%). However, the false-positive results for the Maccura kit were not the first to occur. Tembo et al. conducted a diagnostic validation study of multiple SARS-CoV-2 kits. They evaluated the detection of the Maccura kit using the RealStar SARS-CoV-2 RT-PCR kit as the gold standard and showed that there were 5 false-positive samples among the 18 samples diagnosed as SARS-CoV-2 positive by the Maccura kit, with a false-positive rate of 27% [27]. A higher false-positive rate than ours was observed in the above study. The possible reasons for the false-positive results of the Maccura kit in this study were: 1) The lower limit of detection differed slightly between kits. 2) There were differences in the primers and probes used in different kits [28]. 3) The Maccura kit’s more comprehensive assay design allowed it to detect a broader range of SARS-CoV-2 variants. We checked the announcement of SARS-CoV-2 variant detection status released by two companies (Maccura Biotechnology and Sansure Biotechnology) and found that the Maccura kit could detect more SARS-CoV-2 variant strains (B.1.526 Iota, B.1.429/427 Epsilon, P.2 zeta, etc.) [29, 30]. 4) The Maccura kit targets three different genes (*ORF1ab*, *N*, and *E*), whereas the Sansure kit only targets two genes (*ORF1ab* and *N*). Compared to the *ORF1ab* and *N* genes, the *E* gene is less specific because it has 93.5% sequence homology with other coronaviruses (Sarbecovirus SARS-CoV) [31]. It has also been shown that *E* gene mutations could affect the detection efficiency of the kits [32]. 5) This study was conducted in an emergency situation (mass population screening in a high prevalence area) and did not take samples for nucleic acid integrity testing by sequencing or viral culture. It was possible that virus fragments with no infectious capacity were detected, resulting in false-positive results.

In this study, the new cut-off Ct values were used only for the *ORF1ab* and *N* genes of the Maccura kit, and the test result was considered positive based on the new criteria (*ORF1ab* gene: Ct ≤ 35.00; *N* gene: Ct ≤ 35.07). The validity and the feasibility of the new cut-off values were confirmed by the validation cohort. We did not introduce a cut-off value for the *E* gene because the *ORF1ab* and *N* genes in SARS-CoV-2 are longer with more variant sites [33]; The *N* gene has a high transcript level which will lead to higher sensitivity in the diagnosis [34]. Besides, the *ORF1ab* and *N* genes are recommended as targets of detection in the “Technical guidelines for 2019-nCoV nucleic acid screening with 10-in-1 mix samples” issued by the National Health Commission of the People’s Republic of China [35].

The COVID-19 pandemic has brought a great challenge to many resource-limited areas [36]. Each laboratory should consider its facility, specimen volume, staffing, and financial situation to reasonably procure high-quality results from the test kits. Currently, many studies have compared and analyzed the diagnostic efficacy of different commercially available kits, but most have used characteristic reference substances or the number of cases including COVID-19-positive patients is not sufficient [14, 37, 38]. Yang et al. conducted performance validation of five SARS-CoV-2 NAT kits using characteristic reference substances and found that all kits passed the performance validation except Kinghawk, which failed to detect the detection limit of 500 copies/mL [14]. Lu et al. and Wu et al. evaluated the sensitivity, specificity, positive predictive value, and negative predictive value of multiple SARS-CoV-2 NAT kits using samples from 18 and 26 COVID-19-positive patients, respectively, and their studies provided information on detection protocols in the COVID-19 pandemic [9, 37]. The advantage of this study compared to previous studies was the inclusion of a large real clinical sample (1118 COVID-19-positive patients) to evaluate the practical clinical application of the two kits. On the basis of this, we investigated the difference, correlation, and consistency in the test results between the two kits. We proposed solutions for the improvement of the kits by recalculating the new cut-off Ct values of the Maccura kit and including 163 clinical samples for large-scale validation. Ideally, laboratories should use both NAT kits to fully pursue the accuracy in practical applications. According to the compatibility of the test results with clinical practice, a suitable and cost-effective NAT kit is reasonably needed in the laboratory, or the purchased kits should be improved to minimize false positives or false negatives in practice.

It is worth noting that there are still some limitations in our study. First, the results of NAT are strongly affected by specimen quality, transportation, storage condition, laboratory environment, and operating techniques of testing personnel. It is too difficult to ensure that the testing process is completely consistent for all the samples. Second, the Sansure kit assay used in this study cannot represent the gold standard and may limit the validity of the results. Third, we did not perform additional sequencing and viral culture of false-positive samples for our own reasons (including embarrassment of time, human and material resources) and environmental impacts (emergency screening of large populations in highly endemic areas). Finally, this study has geographical limitations (data collected only in Jilin Province, China) and cannot be scaled up to make more general conclusions.

## Conclusion

To conclude, we analyzed the difference in the testing performance between two SARS-CoV-2 NAT kits using a large number of clinical samples. Some false-positive cases were discovered from the results provided by the Maccura kit, and the cut-off Ct values of the kit were adjusted to improve the accuracy. After re-evaluating the results, the new cut-off values were considered to be applicable in actual clinical work, providing a reference for other laboratories to select and optimize the kits.

## Data Availability

The datasets used and/or analysed during the current study are available from the corresponding author on reasonable request.

## Abbreviations

SARS-CoV-2: Severe acute respiratory syndrome coronavirus 2
COVID-19: Coronavirus disease 2019
NAT: Nucleic acid testing
RT-PCR: Reverse transcription-polymerase chain reaction
NAE: Nucleic acid extraction
Ct: Cycle threshold
SD: Standard deviation
ROC: Receiver operating characteristic
AUC: Area under curve
CI: Confidence interval
